# Beyond conservation: the landscape of chloroplast genome rearrangements in angiosperms

**DOI:** 10.1111/nph.70364

**Published:** 2025-07-04

**Authors:** Luiz Augusto Cauz‐Santos

**Affiliations:** ^1^ Department of Botany and Biodiversity Research University of Vienna 1030 Vienna Austria

**Keywords:** angiosperms, chloroplast genome, gene loss, inversions, IR boundary shifts, plant adaptation, plastome rearrangements, structural variants

## Abstract

Chloroplast genomes (plastomes) have long been considered structurally conserved, but recent sequencing efforts have uncovered pervasive rearrangements that challenge this assumption. This review catalogues the main types of plastome modifications: large and small inversions; insertions and deletions (indels); gene and intron losses; horizontal gene transfers; shifts in inverted repeat boundaries; and gene duplications. It then explains the molecular processes that generate these changes, from repeat‐mediated recombination and slipped‐strand mispairing to rare foreign‐DNA integration events. These structural variants serve as informative phylogenetic markers, enabling resolution of both ancient divergences and recent radiations within angiosperms. Beyond their value for systematics, plastome rearrangements can reshape gene order and copy number, with measurable effects on gene expression, metabolic pathways, and photosynthetic efficiency. Evidence shows that, in certain lineages, plastid genes have been transferred to the nucleus to compensate for gene loss and preserve essential cellular functions. Looking ahead, three emerging approaches promise to deepen our understanding of plastome dynamics: comprehensive pan‐plastome surveys coupled with long‐read sequencing of under‐sampled lineages; targeted plastid transformation to engineer specific rearrangements; and advanced genome editing to test their adaptive significance. Together, these strategies will illuminate how plastid structural change impacts plant evolution and adaptation.

## Introduction: chloroplast genome architecture and variability

**Fig. 1:**
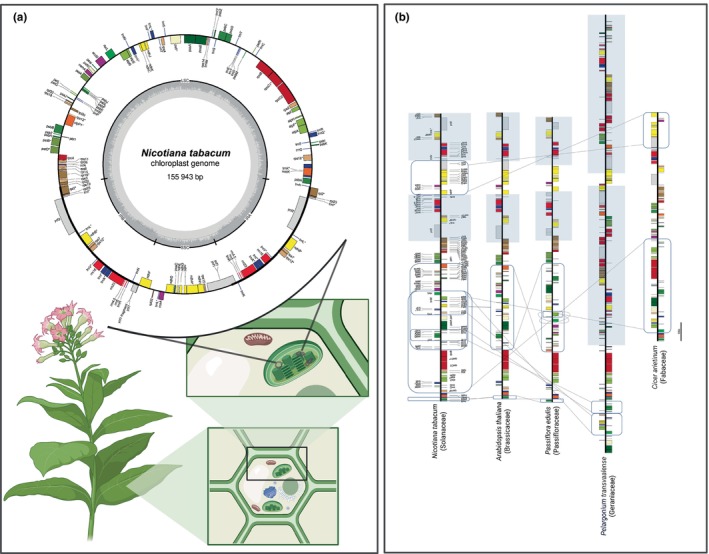
Overview of chloroplast genome structure and comparative synteny among angiosperms. (a) A circular map of the *Nicotiana tabacum* chloroplast genome (GenBank accession NC_001879) illustrates the canonical quadripartite structure found in most angiosperm plastomes, comprising a large single copy (LSC) region, a small single copy (SSC) region, and two inverted repeats (IRs). Genes are color‐coded by functional category and their orientations are indicated. (b) Comparative synteny between *N. tabacum* (NC_001879) and four other angiosperms: *Arabidopsis thaliana* (NC_000932), *Cicer arietinum* (NC_011163), *Passiflora edulis* (NC_034285), and *Pelargonium transvaalense* (NC_031206). Notably, *C. arietinum* serves as the representative of the IR‐lacking clade (IRLC), exemplifying the loss of the typical inverted‐repeat structure. Structural rearrangements, such as inversions, are indicated by dashed connecting blocks, and the inverted‐repeat region is shaded in blue. Figure was partially created in BioRender (https://biorender.com/sghsas8).

Angiosperms, the flowering plants, are among the most diverse and dynamic groups in the plant kingdom, and their organellar genomes offer critical insights into this complexity. The study of the organellar genomes in this clade advanced significantly with the advent of complete sequencing of chloroplast (cp) genomes, often referred as plastome or plastid. An important milestone was the sequencing of the tobacco chloroplast genome (*Nicotiana tabacum* L.) in 1986, which provided a foundational blueprint for understanding chloroplast organization and gene expression in angiosperms (Shinozaki *et al*., 1986). Typically, angiosperm chloroplast genomes are circular, with a quadripartite structure comprising two inverted repeats (IRs) separating a large single‐copy from a small single‐copy (SSC) region (Fig. 1a), usually spanning 120–160 kb and encoding 110–130 unique genes. Exceptions exist: for example, *Pelargonium transvaalense* R. Knuth has an expanded plastome of 242 kb due to large IR expansions (Weng *et al*., 2017), while the parasitic species *Rafflesia lagascae* Blanco may lack a recognizable plastome altogether, with only degraded gene fragments detected despite deep sequencing (Molina *et al*., 2014). Genes for rRNA and tRNA generally reside within the IRs, while protein‐coding genes, essential for photosynthesis and metabolism (Table 1), are located in the single‐copy regions.

**Table 1:** Functional categories of plastidencoded genes and their biological processes.

Functional category	Genes included	Primary function
Photosynthesis and electron transport	*psaA*–*psaM* (Photosystem I), *psbA*–*psbZ* (Photosystem II), *petA*–*petN* (cytochrome *b*_*6*_ *f* complex), *atpA*–*atpI* (ATP synthase), *ndhA*–*ndhK* (NDH complex), *rbcL*	Light reactions, electron transport, carbon fixation, ATP synthesis
Transcription and RNA processing	*rpoA*, *rpoB*, *rpoC1*, *rpoC2* (PEP subunits); *rrn16*, *rrn23*, *rrn5*, *rrn4.5* (rRNAs); *c*. 30 tRNAs	Plastid gene transcription and RNA maturation, including rRNA and tRNA processing
Translation (ribosomal proteins and initiation)	*rps2*, *rps3*, *rps4*, *rps7*, *rps8*, *rps11*, *rps12*, *rps14*, *rps15*, *rps16*, *rps18*, *rps19*; *rpl2*, *rpl14*, *rpl16*, *rpl20*, *rpl22*, *rpl23*, *rpl32*, *rpl33*, *rpl36, infA* (translation initiation factor)	Ribosome assembly, translation initiation and protein synthesis
Housekeeping/metabolism	*accD* (acetyl‐CoA carboxylase), *clpP* (protease), *ycf1*–*ycf4* and other *ycf* genes (conserved hypothetical proteins)	Fatty‐acid metabolism, protein turnover, essential conserved functions
Other genes	*ccsA* (cytochrome *c* synthesis); *cemA* (envelope membrane protein); *matK* (maturase)	Maturation of cytochrome *c*, envelope membrane transport, and splicing of group II introns in plastid transcripts

Early studies employing restriction site mapping revealed unexpected variations, such as the loss of a typical IR in pea (Palmer & Thompson, 1981), challenging the long‐held assumption of a conserved plastid genome structure. Subsequent comparative analyses uncovered a spectrum of structural modifications, including large inversions, IR boundary shifts, gene and intron losses, and duplications (Jansen & Palmer, 1987; Cosner *et al*., 2004). What are the molecular mechanisms responsible for driving these rearrangements? How do such structural changes affect gene expression and photosynthetic performance? And how might they contribute to broader evolutionary processes such as adaptation and speciation? These questions lie at the heart of a dynamic research field that rapidly evolved as new genomic technologies emerged.

Advances in next‐generation sequencing have transformed our understanding of chloroplast genome evolution in angiosperms by exposing extensive structural variability within and among species. Landmark studies in groups such as Cactaceae, Geraniaceae, Passifloraceae, Plantaginaceae, Onagraceae, and the monocot order Poales have demonstrated that plastomes are far more reconfigured than once believed (Greiner *et al*., 2008; Guisinger *et al*., 2011; Weng *et al*., 2014; Cauz‐Santos *et al*., 2020; Yu *et al*., 2023; J. Wang *et al*., 2024; Wu *et al*., 2024). Emerging long‐read sequencing and pan‐plastome approaches are now revealing even subtler structural variants, prompting a reexamination of how these rearrangements influence key physiological processes and drive evolutionary diversification. This review synthesizes insights into the structural variability of angiosperm chloroplast genomes, the mechanisms driving these rearrangements, and their evolutionary and functional consequences.

## Types and mechanisms of rearrangement

Chloroplast genomes exhibit a variety of structural rearrangements driven by distinct molecular mechanisms (Table 2). Among the major classes of rearrangements, inversions represent one of the most common and well characterized forms of structural change.

**Table 2:** Major types of plastome rearrangements and their underlying mechanisms.

Rearrangement type	Description
Inversion	Segment reversed in orientation via recombination between repeats or microhomology
Insertion/deletion	Gain or loss of sequence, often in noncoding regions, mediated by slippedstrand mispairing or hairpin loops
IR boundary shift	Expansion or contraction of inverted repeat boundaries driven by gene conversion or recombination at IR/SC junctions
Gene loss or transfer	Loss of plastid genes, sometimes complemented by functional nuclear‐encoded copies
Duplication	Copying of genes or regions due to transposition or IR‐mediated expansion
Horizontal DNA transfer	Integration of mitochondria or other foreign DNA into the plastome

IR, inverted‐repeat.

### Inversions

**Fig. 2:**
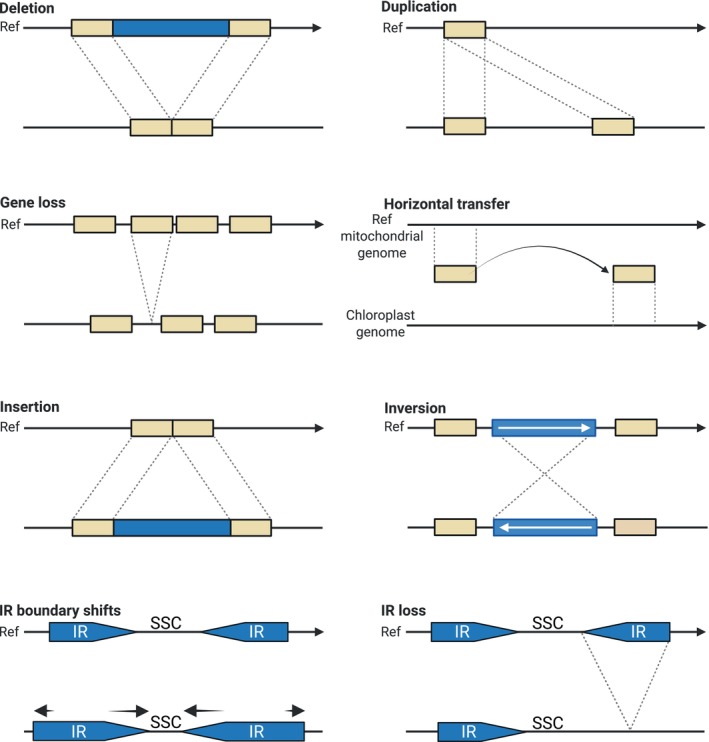
Schematic overview of chloroplast genome rearrangement types. This schema illustrates a representative chloroplast genome in its reference state (Ref) and summarizes key structural modifications. Deletions are represented as missing segments relative to the reference, while duplications appear as repeated sequence blocks. Gene loss is indicated by the absence of specific genes that are normally present in the reference genome. Horizontal gene transfer events are shown by arrows indicating the movement of sequences from the mitochondrial genome to the plastome. Inversions are depicted as segments that have been excised and reinserted in reverse orientation, thereby altering gene order. Insertions are illustrated as additional sequence blocks that disrupt the typical organization. Finally, inverted repeat (IR) boundary shifts are indicated by arrows outside the boxes, reflecting the sequence expansion at the IR large single‐copy (LSC)/small single‐copy (SSC) junctions. The figure was created in BioRender (https://biorender.com/hh2dsow) and is inspired by the conceptual schema from Alkan *et al*. (2011).

Inversions in the structure of chloroplast genomes occurs when a DNA segment is excised and reinserted in reverse orientation (Fig. 2), often mediated by short direct or inverted repeats (Ogihara *et al*., 1988). For example, in legumes a 78 kb inversion in the large single‐copy region has been proposed to account for multiple rearrangements in pea cpDNA, with inversion endpoints frequently located in tRNA‐rich spacer regions (Palmer *et al*., 1988), while studies in wheat and *Aegilops* L. showed that short direct repeats could induce both deletions and inversion rearrangements (Ogihara *et al*., 1988). In rice, several inversions in the large single‐copy region, accompanied by the appearance of a chimeric tRNA pseudogene at an inversion endpoint, have indicated illegitimate recombination between tRNA genes (Hiratsuka *et al*., 1989). Gene mapping in subclover uncovered eight large inversions and additional complex rearrangements mediated by dispersed repeats, providing early evidence of transposable element involvement in plastid genomes (Milligan *et al*., 1989). More recent studies have further underscored the role of repetitive sequences in driving inversion events. In particular, analyses in Fabaceae within the inverted repeat lacking clade (IRLC) have revealed that repeat‐mediated illegitimate recombination is potentially the major mechanism leading to extensive genomic rearrangements, even when an IR reemerges (Wu *et al*., 2021). Additionally, work in *Astragalus* L. has demonstrated that short repeats near inversion endpoints could be resulted from microhomology‐mediated break‐induced replication (Charboneau *et al*., 2021).

Inversions have been widely reported as an important mechanism driving cp genome evolution across diverse lineages, as illustrated by examples in Geraniaceae (Guisinger *et al*., 2011; Weng *et al*., 2014) and Passifloraceae (Cauz‐Santos *et al*., 2020). Additionally, small inversions (SIs) are also pervasive in angiosperm chloroplast genomes, typically ranging from 4 to 50 bp in length, and are likely facilitated by hairpin secondary structures (Kelchner & Wendel, 1996; Catalano *et al*., 2009). Collectively, large and SIs demonstrate that chloroplast genomes are subject to a range of structural rearrangement events, each contributing to the overall genomic dynamism and potentially influencing gene regulation and evolution.

### Insertions/deletions (Indels)

Indels are key drivers of chloroplast genome evolution and serve as robust markers for phylogenetic and population studies. These insertion and deletion events occur primarily in noncoding regions, where they often result in observable size polymorphisms that are invaluable for differentiating among closely related taxa and assessing intraspecific diversity.

Indels typically arise via slipped‐strand mispairing: during DNA replication, repetitive sequences can misalign, forming a loop in the newly synthesized strand. This loop may result in the addition (insertion) or loss (deletion) of repeat units. At the same time, hairpin structures in AT‐rich regions may promote similar mispairing and strand slippage (Kelchner & Wendel, 1996).

Recent research has demonstrated the applications of chloroplast indel markers in agricultural and ecological research. For example, Fang *et al*. (2021) assembled the chloroplast genomes of two jute species, *Corchorus capsularis* L. and *Corchorus olitorius* L., identifying 294 indel sites. Among these, one marker (cpInDel 205) effectively distinguished between the two species, thereby demonstrating its utility for species discrimination and as a tool for molecular marker development in crop improvement programs. In maize, R. Wang *et al*. (2024) used next‐generation sequencing to develop a Varietal Chloroplast Panel based on single nucleotide polymorphisms (SNPs) and InDels, which effectively characterized maternal inheritance traits such as cytoplasmic male sterility, which is a critical factor for hybrid breeding.

### Horizontal transfers

Occasionally, horizontal transfers lead to more complexity in cp genome architecture. Although plastomes are generally resistant to foreign DNA integration, several events of mitochondrial‐to‐plastid DNA transfer have been documented. For example, the cp genome of *Convallaria keiskei* Miq. harbors a *c*. 3.3 kb segment of mitochondrial DNA containing a pseudogenized *rpl10* gene, a unique feature within Asparagaceae (Raman *et al*., 2021). Similar transfers have been reported in bamboo, with the plastid IR region featuring a *c*. 2.7 kb insertion (Ma *et al*., 2015), and a recent study in Apiaceae uncovered partial mitochondrial genome fragments within the plastome of Ferulinae lineages (Jo *et al*., 2024).

### Gene loss

Chloroplast genomes also evolve by losing specific genes and introns, a process that often request compensatory mechanisms to preserve vital functions. For example, the gene *infA*, encoding translation initiation factor 1, is intact in most angiosperms but has been lost from *c*. 24 lineages (including nearly all Rosids), sometimes accompanied by functional nuclear copies (Millen *et al*., 2001). In *Corydalis* DC. (Papaveraceae), the loss of *accD* appears to be compensated by a functional copy relocated to the nuclear genome, which shows evidence of expression, indicating successful integration. Meanwhile, the NADH‐plastoquinone oxidoreductase (*ndh*) complex was concurrently lost from both nuclear and plastid genomes (Park *et al*., 2024). Gene loss is especially pronounced in parasitic plants. For example, in the holoparasitic Lennoaceae, nearly half of the plastid genes are lost or pseudogenized, only 60 of the original 114 genes are retained, mainly those involved in housekeeping functions, while all photosynthesis‐related genes are absent (Schneider *et al*., 2018).

Experimental systems have provided insight into the mechanisms involved in the transfer of genetic material between chloroplast and nucleus. For instance, functional gene transfer has been reconstructed, showing that integrated cpDNA segments can acquire promoters and proper RNA processing signals (Stegemann & Bock, 2006). Additionally, Fuentes *et al*. (2012) demonstrated that even intron‐containing chloroplast genes can be transferred via DNA‐mediated events, with cryptic splice sites ensuring correct mRNA processing.

### Duplications

Plastid gene duplications outside of the IR are uncommon and may arise through mechanisms distinct from those driven by IR expansion, such as duplicative transposition (Haberle *et al*., 2008). For example, in *Trachelium caeruleum* L., the duplication event involves not only a protein‐coding gene but also an associated tRNA gene, with the genes *psbJ* and *trnI‐CAU* co‐duplicated (Haberle *et al*., 2008). This example illustrates that even small, functionally linked genomic segments can be subject to such duplicative events. In addition, the plastomes of Geraniaceae are characterized by multiple gene duplications as a consequence of extensive structural rearrangements (Guisinger *et al*., 2011).

### IR boundary shifts

Inverted repeat boundary shifts, expansions, and contractions are key mechanisms driving plastome size variation and structural diversity. Variations in IR endpoints largely result from gene conversion and recombination; for example, in *Nicotiana acuminata* (Graham) Hook., a double‐strand break and subsequent recombination between poly(A) tracts in the *clpP* intron and upstream of *rps19* led to an IR expansion of over 12 kb (Goulding *et al*., 1996). Similarly, the IR in *P. transvaalense* reaches up to 87 724 bp, the largest observed among angiosperms (Weng *et al*., 2017). By contrast, extreme cases are observed in Cactaceae, where the IR in *Frailea castanea* var. *nitens* contracts to 437 bp (Yu *et al*., 2023), a pronounced deviation from the IR sizes typically observed in angiosperms. Similarly, within *Euphorbia* L., extreme variations in IR size have been reported, with some species possessing IRs as short as 355 bp compared to others that retain an IR of up to 43 573 bp (Wei *et al*., 2021). These examples highlight the remarkable plasticity of the chloroplast genome under different evolutionary pressures.

Additionally, dryland plants in the family Zygophyllaceae exhibit significant IR contractions, with reductions on the order of 16–24 kb. This marked decrease in IR length not only results in a reduced overall plastome size but is also associated with the loss of genes typically maintained within the IR regions (X. Wang *et al*., 2022). These examples highlight the wide spectrum of IR boundary variations across angiosperms. Such variations have important implications for plastome stability and evolution, influencing overall genome organization and gene expression. The expansion of the IR into the single‐copy regions results in the incorporation and duplication of genes that are normally present as single copies. This phenomenon not only increases the overall plastome size but also affects gene dosage and regulation (Krämer *et al*., 2024). Overall, these IR boundary shifts illustrate how expansions and contractions can reshape plastome architecture, influence gene content and dosage, and contribute to lineage‐specific patterns of genome evolution.

### IR loss

Complete IR loss is a rare but notable event in plastome evolution. Early work suggested that IR regions contributed to chloroplast stability, with IR loss correlating with increased rearrangements (Palmer & Thompson, 1982). However, later studies indicate that IR loss is one among several rearrangements that often occur alongside other structural changes rather than driving instability alone (Sabir *et al*., 2014; Z.‐X. Wang *et al*., 2022). Comparisons among legumes reveal diverse IR configurations, including complete absence of the typical IR, as seen in the IRLC within Fabaceae (Palmer *et al*., 1987). Complete IR loss has also been documented in other species, such as *Erodium texanum* A. Gray (Geraniaceae; Guisinger *et al*., 2011), in certain *Passiflora* L. species (Cauz‐Santos *et al*., 2020), and in the Poales species *Xyris capensis* Thunb. (Wu *et al*., 2024). Collectively, these examples demonstrate that IR loss typically occurs in concert with other genomic rearrangements and is not, by itself, the primary cause of plastome instability.

## Heteroplasmy and structural isoforms

Although plastomes have long been treated as a single, uniform molecule per individual, advances in sequencing have revealed pervasive structural heteroplasmy, the coexistence of multiple plastome arrangements within a single organism. In a landmark study, Lee *et al*. (2020) combined PacBio and Illumina data to show that two *Eleocharis* R.Br. species (Cyperaceae) harbor at least four distinct plastome structural types, with repeats up to *c*. 5 kb mediating inversions and other rearrangements. Extending this pattern to a broader taxonomic scale, a recent order‐wide analysis of Poales (93 plastomes across 16 families) revealed widespread structural heteroplasmy, diversified inversions (up to 13 per individual), rare IR changes, and repeat‐linked rearrangements even within single accessions (Wu *et al*., 2024).

## Evolutionary implications and functional consequences

Collectively, the diverse rearrangement types underscore the dynamic evolution of chloroplast genomes in angiosperms. The structural changes, ranging from inversions and indels to gene loss and IR boundary shifts, not only serve as robust phylogenetic markers but also have important implications for plastid function. By reshaping gene order and copy number, these alterations can directly influence gene expression patterns, metabolic pathways, and photosynthetic performance. Moreover, such genomic reconfigurations create novel selective pressures and opportunities for adaptation, contributing to evolutionary divergence among plant lineages.

Rearrangement events, such as inversions, have been widely used in phylogenetic studies (Palmer *et al*., 1988; Cosner *et al*., 2004). For example, in Leguminosae, a 50 kb inversion marks a clade within the Papilionoideae that distinguishes it from the Mimosoideae and Caesalpinioideae (Doyle *et al*., 1996). In Asteraceae, a combination of a 22 kb inversion and a smaller 3.3 kb inversion has defined major evolutionary splits, with divergence estimates dating these events to the late Eocene (Jansen & Palmer, 1987; Kim & Lee, 2005). Rearrangement patterns observed in Campanulaceae and core Tillandsioideae (Cosner *et al*., 2004; Vera‐Paz *et al*., 2022), have likewise reinforced phylogenetic and taxonomic hypotheses, highlighting the broad utility of chloroplast genome structural variation for systematic studies.

However, some rearrangements, such as IR loss, could have profound evolutionary and functional implications for chloroplast genomes. Experimental removal of the IR from the tobacco plastid genome demonstrated that the IR enhances translation capacity by providing a duplicated ribosomal RNA operon, as evidenced by a mild reduction in plastid ribosome number in IR‐deleted plants. These plants compensate with increased plastid genome copy numbers, which in this study were estimated using quantitative polymerase chain reaction measurements (Krämer *et al*., 2024). Early studies had suggested that the presence of a large IR might contribute to chloroplast genome stability. For example, Palmer & Thompson (1982) exploring seven angiosperm plastomes observed that species with a complete IR exhibited a stable gene order, while legumes lacking an IR segment displayed extensive rearrangements. However, later studies challenged this view, reporting that the deletion of a complete IR does not necessarily lead to genome instability or trigger additional rearrangements (Palmer *et al*., 1987). Instead, IR loss appears to be just one of several types of rearrangements (Sabir *et al*., 2014). In *Erodium*, plastome stability appears to be unaffected by whether the IR is present or absent (Blazier *et al*., 2016).

Comparative analyses further reveal that large‐scale IR expansions and contractions can affect nucleotide substitution rates in ways that go beyond the simple presence or absence of the IR (Weng *et al*., 2017). Genes relocated into expanded IR regions often exhibit rate heterotachy, variation in evolutionary rates among genes and lineages, a well‐documented phenomenon in plastid genomes (Wu *et al*., 2011). These structural and population‐level processes have important functional implications: modifications in IR size can influence gene dosage and the expression of rRNA and adjacent genes, thereby impacting chloroplast ribosome assembly, photosynthetic efficiency, and plant stress responses. Such multilayered variation raises key questions about how chloroplast genome restructuring drives adaptive evolution and speciation in natural plant populations.

A recent study by Li *et al*. (2025) highlights an even more intricate layer of plastome dynamics: interplastomic recombination. Through sliding‐window phylogenetic analysis of the disjunct genus *Hedyosmum* Sw., they detected mosaic plastomes in *Hedyosmum orientale* Merr. & Chun, whose plastid genome appears to have originated via recombination between two diverged ancestral plastomes, one from the subgenus *Hedyosmum* and the other from the subgenus *Tafalla* Ruiz & Pav. This mosaic pattern challenges the convention of treating plastomes as a uniform, non‐recombining locus, a practice that may mask hidden genomic variation.

The structural alterations have important implications for plant function and adaptation, for instance, modifications in IR size may influence gene dosage and the expression levels of rRNA and neighboring genes, affecting the assembly and performance of the chloroplast ribosome, photosynthetic efficiency, and stress responses. Such changes could ultimately shape plant physiological performance under varying environmental conditions, driving adaptive evolution and even speciation. Such changes raise important questions regarding adaptive evolution: To what extent do alterations in chloroplast genome structure drive plant adaptation to diverse environmental conditions?

It is important to recognize that structural rearrangements also have profound implications for gene sequence evolution. For instance, extensive rearrangements are often associated with accelerated amino acid substitution rates in key chloroplast genes. Blazier *et al*. (2016) found that in lineages with extensive rearrangements, such as in some Geraniaceae, divergent *rpoA* open reading frames are maintained under purifying selection, suggesting that illegitimate recombination is one of the drivers of their sequence evolution. Similarly, accelerated evolution of *clpP*, accompanied by gene duplications and intron losses, has been documented in multiple lineages (Williams *et al*., 2019), indicating that structural changes can influence the rate of protein evolution. In *Hypericum* L., dynamic plastome rearrangements, including multiple inversions, IR boundary shifts, and gene relocations, are associated with elevated substitution rates and even signatures of positive selection in several genes (Claude *et al*., 2022). Additionally, broad comparative analyses reveal that the degree of structural rearrangement is significantly correlated with increased nonsynonymous substitution rates, supporting the idea that genomic reorganization can alter selective pressures on plastid genes (Weng *et al*., 2014). Together, these findings demonstrate a clear connection between plastome reconfiguration and the molecular evolution of chloroplast genes, shedding light on how these changes may contribute to plant adaptation and diversification.

Changes in chloroplast genome structure can significantly impact gene expression and metabolic performance. For example, deletion of *ycf4* in tobacco severely disrupts the assembly of photosynthetic complexes and impairs photoautotrophic growth (Khan *et al*., 2022). Other plastid gene deletions also have critical functional consequences; for instance, mutations in the chloroplast *rps5* gene in *Arabidopsis thaliana* (L) Heynh. impair 16S rRNA processing, reduce the expression of core photosystem components, and compromise cold tolerance. Additionally, *rps2*, *rps4*, and *rpl20* have been shown to be essential for ribosome function and plastid development, as knockout lines exhibit stable heteroplasmy and morphological defects typical of essential gene disruptions (Rogalski *et al*., 2008; Zhang *et al*., 2016). In many cases, loss of key plastid genes is compensated by their transfer to the nucleus. In the Campanulaceae, the plastid *accD* gene, critical for fatty acid biosynthesis, has been relocated to the nucleus, where it produces a truncated yet active protein (Rousseau‐Gueutin *et al*., 2013). Similarly, in *Passiflora*, the transfer of *rps7* maintains vital chloroplast functions despite extensive rearrangements and gene losses (Shrestha *et al*., 2020). These findings reveal the complexity of plastid genome reconfiguration and its profound impact on cellular function. They underscore how plastid to nucleus gene transfer serves as an important compensatory mechanism to safeguard essential metabolic processes, thereby maintaining plant viability in the face of genomic disruptions.

## Unraveling plastid architecture with long‐read sequencing

Recent developments in long‐read sequencing have redefined our capacity to analyze chloroplast genomes, illuminating intricate structural rearrangements that often remain hidden when using short‐read techniques. Long‐read platforms, such as those provided by Oxford Nanopore Technologies (ONT) and Pacific Biosciences, generate reads that span extensive repeat regions, allowing researchers to capture complete genomic segments and accurately resolve regions that are problematic for short‐read assemblers. A compelling example of this technological advantage can be found in studies of *Passiflora*. In this genus, the loss of one IR in *Passiflora costaricensis* was conclusively validated by long‐read sequencing (Cauz‐Santos *et al*., 2020). This breakthrough not only underscores the efficiency of long‐read methodologies in accurately demarcating repeat‐mediated breakpoints but also highlights significant variation in plastome structure among closely related species.

Additionally, the use of hybrid sequencing strategies, combining the high per‐base accuracy of Illumina reads with the long‐range continuity offered by ONT, has facilitated the detection of unexpected horizontal transfer events. For instance, recent hybrid assemblies have identified insertions of mitochondrial DNA into plastomes, a phenomenon that might be overlooked without the benefit of extended read lengths (Jo *et al*., 2024). Another example using a hybrid assembly of Illumina and ONT data for the assembly of complete plastid and mitochondrial genomes of *Corydalis pauciovulata* Ohwi revealed unusual features such as an expanded IR (46 060 bp) and a miniaturized SSC (202 bp) region in the cp genome, while also documenting significant gene losses specific to the plastid compartment (Park *et al*., 2024).

Complementing these advances in sequencing is the development of specialized bioinformatics pipelines, such as the ptGAUL tool (Zhou *et al*., 2023), designed specifically to tackle the challenges associated with long‐read data. By rapidly identifying large repeats, inversions, and gene transfers with relatively low sequencing coverage (*c*. 50×), such pipelines streamline plastome assembly and ensure greater accuracy in variant detection.

## Conclusions and future perspectives

The rapid evolution of long‐read sequencing and pan‐genomic approaches offers exciting new avenues for uncovering the full spectrum of plastome rearrangements and their evolutionary implications. In the near future, comprehensive pan‐plastome analyses will likely become central to understanding the structural diversity and population dynamics of plastid genomes in angiosperms. Unlike SNP‐focused approaches, pan‐plastome strategies are particularly well suited to uncover larger‐scale variation such as gene duplications, inversions, IR boundary shifts, and structural polymorphisms across lineages. A pioneering example is the study by Magdy *et al*. (2019), which applied a pan‐plastome approach to over 300 accessions of cultivated *Capsicum* L. species, successfully resolving taxonomic ambiguities and identifying informative structural markers. More recent work has extended this approach to *Hibiscus syriacus* L. using long‐read and short‐read data to detect cultivar‐specific structural variants (Go *et al*., 2024), and to *Medicago sativa* L., where pan‐plastome analyses revealed population structure and differential expression linked to cold stress (Zhang *et al*., 2024).

From an evolutionary perspective, plastome rearrangements can also influence speciation and adaptation. Zupok *et al*. (2021) showed that in evening primrose (*Oenothera* L.), a deletion upstream of the *psbB* operon promoter disrupts the expression of photosynthesis‐related genes under high light, leading to plastid–nuclear incompatibility and the emergence of hybrid phenotypes (e.g. the AB‐I phenotype) that function as reproductive barriers linked to habitat adaptation. In addition, genome transformation techniques are being used to detect and characterize the functional roles of plastid genome rearrangements. Targeted plastid transformation has successfully removed the entire IR region in tobacco, providing valuable insights into the feasibility of designing minimal‐size synthetic plastid genomes (Krämer *et al*., 2024), although such transformations are still technically demanding and currently limited to a few model species like tobacco. On the functional side, experiments have shown that transgene insertions can unintentionally disrupt the expression of neighboring native genes (Ghandour *et al*., 2023), underscoring the delicate balance of the plastid genome and the significant metabolic consequences of such structural changes. These findings pave the way for advanced genome editing tools to introduce precise, targeted rearrangements in the plastome, thereby enabling researchers to mimic natural structural variants and investigate their evolutionary impact.

Despite transformative advances in longread sequencing, panplastome analyses, and genome editing that are revealing the full breadth of plastome structural variation and its functional consequences, plastome sampling remains limited. As of June 11, 2025, the NCBI Organelle Genome Dataset contains just 13 761 angiosperm chloroplast genomes, representing only *c*. 4.6% of the *c*. 300 000 flowering plant species. This gap constrains our ability to understand both the diversity of plastome architectures and their ecological and evolutionary roles across angiosperms. Future research should prioritize: (1) longread plastome sequencing across underrepresented angiosperms to improve taxonomic coverage; (2) population‐level panplastome surveys to identify structural variants and their functional impacts; and (3) experimental systems, like plastid transformation or genome editing, to assess the adaptive significance of natural and engineered plastome rearrangements.

## Competing interests

None declared.

## Disclaimer

The New Phytologist Foundation remains neutral with regard to jurisdictional claims in maps and in any institutional affiliations.
